# Replication competent virus as an important source of bias in HIV latency models utilizing single round viral constructs

**DOI:** 10.1186/s12977-014-0070-3

**Published:** 2014-08-21

**Authors:** Pawel Bonczkowski, Ward De Spiegelaere, Alberto Bosque, Cory H White, Anouk Van Nuffel, Eva Malatinkova, Maja Kiselinova, Wim Trypsteen, Wojciech Witkowski, Jolien Vermeire, Bruno Verhasselt, Laura Martins, Christopher H Woelk, Vicente Planelles, Linos Vandekerckhove

**Affiliations:** HIV Translational Research Unit, Department of Internal Medicine, Ghent University and University Hospital, Ghent, Belgium; Division of Microbiology and Immunology, Department of Pathology, University Of Utah School of Medicine, Emma Eccles Jones Medical Research Building, Salt Lake City, UT 84112 USA; Department of Medicine, University of California San Diego, La Jolla, USA; Department of Clinical Chemistry, Microbiology and Immunology, Ghent University, Ghent, Belgium; Faculty of Medicine, University of Southampton, Southampton, UK

**Keywords:** Single round HIV, Recombination, Latency models, Central memory T cells, Non-polarized T cells, HIV latency

## Abstract

The central memory T cell (TCM) model forms a unique HIV-1 latency model based on primary cells that closely resemble in vivo TCM. The virus employed in this model is based on an engineered vector incapable of replication after initial infection. We show that despite this strategy, replication competent viral particles are released into the culture medium due to recombination between overlapping sequences of the env deleted HIV genome that is co-transfected with intact env. This finding emphasizes the need for careful data analysis and interpretation if similar constructs are employed and urges for additional caution during laboratory work.

**Electronic supplementary material:**

The online version of this article (doi:10.1186/s12977-014-0070-3) contains supplementary material, which is available to authorized users.

## Commentary

The recent interest in HIV latency led to the development of cell models recapitulating viral latency in vitro*.* The T_CM_ model published by Bosque and Planelles [1] represents a widely used method. This model, based on in vitro differentiated central memory cells combined with a replication deficient virus, produces high numbers of latently infected primary cells. Here, we show that the viral construct used in this model can become replication competent due to recombination. We observed spreading infection and high variability between repeated experiments using this T_CM_ model (Additional file 1: Figure S2). This urges a re-analysis of the data and poses important biosafety concerns on the use of similar viral constructs.

**Figure 1:**
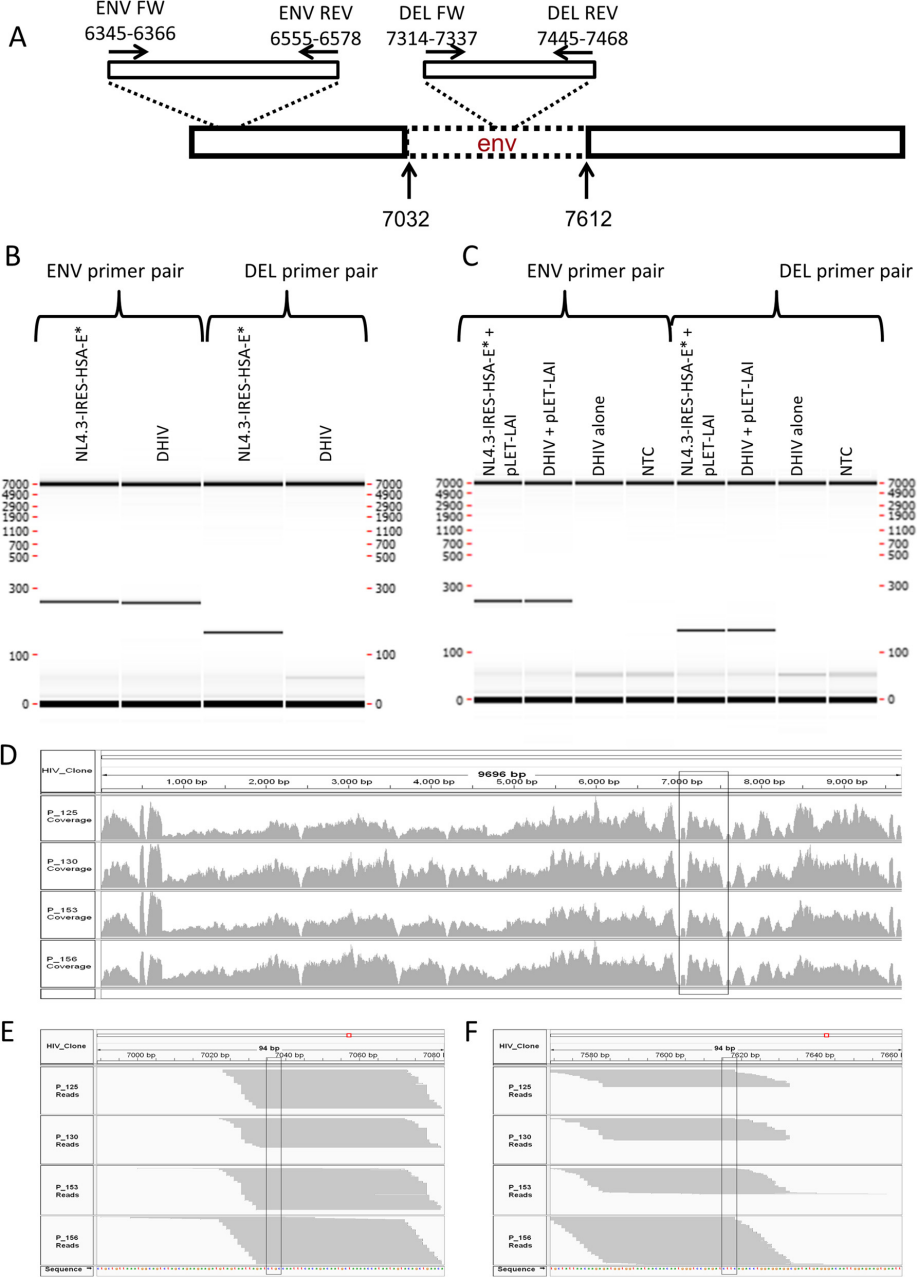
**PCR and NGS-based confirmation of recombination between the DHIV and pLET-LAI constructs as a source of replication-competent virus.** To generate a vector capable of a single round of replication only, the vector employed in the original TCM model is produced with the env-deficient DHIV construct co-transfected with intact env. Recombination between overlapping sequences of these plasmids restores an intact env sequence in the viral genome and produces replication-competent virus. (cfr. Additional file 1). **A-C**. PCR based data A. Primer pairs used in the study aligned to the envelope gene of DHIV construct. Primer pair ENV aligns to the common sequence of env of DHIV and of the full length env, primer pair DEL aligns to the intact env sequence within the deleted part (dotted line) of DHIV. **B-C**. Electrophoretic separation of PCR products performed on plasmid DNA-NL4.3-IRES-HSA-E* and DHIV **(B)** and on DNA from cells infected with the indicated viruses **(C)**. The positive signal in cells infected with DHIV + pLET-LAI indicates the presence of a full length env sequence in the viral DNA. **D-F**. Expression of HIV RNA in the latent TCM model derived from RNA-Seq analysis of 4 donor cells infected with DHIV + pLET-LAI. **D**. The plot representing the number of times individual nucleotides were mapped to the HIV genome. The delineated region indicates the env region deleted in the original DHIV. The high number of mapped reads indicates that the complete env sequence is expressed. **E-F**. The plot representing reads spanning the deletion in DHIV env region at the beginning **(E)** and end **(F)** of the deletion. Collectively, the electrophoretic analysis of integrated proviral DNA and the alignment of NGS reads in the region originally containing a deletion in env indicate that an intact sequence of env was restored in the construct.

The viral construct originally employed was produced with an HIV-1NL4.3-derived viral vector-DHIV-containing a 600 bp deletion in the *env* reading frame. This plasmid is co-transfected with pLET-LAI-a plasmid containing the wild type *env* sequence from HIV-1_LAI_. Our data indicate that the viral supernatant consists of a mix of single round vectors and replication competent viruses, generated by recombination between the *env* sequences (Figure 1, Additional file 1).

These results may not be surprising, considering the stringent precautions taken in the field of lentiviral transduction, requiring three or four (up to 7 a in super-split system [2]) separate plasmids for co-transfection [3,4]. However, two vector constructs are still often used in basic HIV research. The current findings emphasize the biosafety concern for laboratories working with vectors that are assumed to be replication deficient, but can become fully competent viruses. Moreover, these findings urge a re-analysis of the published data derived from the T_CM_ model as well as from data using similar constructs.

In the original paper describing the T_CM_ model [1], NFAT was characterized as the principal transcription factor mediating reactivation of latent viruses after antigen stimulation. Considering the current data, the role of NFAT should be reassessed due to its potential involvement in two different phenomena: reactivation from latency and increasing viral replication kinetics as previously described [5]. Whether NF-κB or NFAT are required for reactivating latent viruses in T_CM_ still needs to be addressed. In accordance to this, the findings that the JAK-STAT pathway [6] or PIM-1 [7] are required for viral replication still hold, but the data cannot discern whether the effect is due to inhibition of reactivation or inhibition of virus replication kinetics.

In contrast to earlier interpretations [8], cellular p24 positive staining after antigen stimulation is not an unequivocal sign of reactivated virus, but rather a combination of that and spreading viral replication. Therefore, the number of latently infected cells should be lower than initially inferred. This is likely to impact deep sequencing and transcriptomic experiments.

A recent study compared integration sites between induced and non-induced proviruses across five latency models, including the T_CM_ model [9]. An *env*(−) virus was used in which *nef* was replaced with GFP. After infection, GFP(−) cells (presumably, latently infected) were sorted and cultured for 9 days prior to integration site analysis [9]. Although the sorting strategy excluded HIV producing cells, a minor fraction of early stage infected cells which did not yet start producing GFP could be present and introduce a bias to the data. The use of an antiretroviral that blocks integration may have provided a quantitative assessment of the extent of this bias.

Interestingly, in the T_CM_ model there is a clear increase in p24 positive cells between samples treated with integrase inhibitors followed by antigen stimulation and samples that are not stimulated. This indicates that a fraction of the infected cells are latently infected despite ongoing replication. Therefore, this model continues to be valuable for HIV latency research. This is supported by results that were corroborated in subsequent or parallel studies with alternative models.

In the study characterizing IL-7-mediated homeostatic proliferation [10], cells were infected with a virus encoding GFP and dividing and non-dividing GFP(−) cells were sorted using a proliferation dye. Both subsets carried integrated HIV DNA and were able to induce viral production after antigen stimulation. The conclusion that cells can undergo homeostatic proliferation in the absence of viral production still holds, especially considering the sorting strategy. This mechanism has been supported by in vivo data [11]. Additionally, the data showing that Pam3CSK4 [12] can reactivate HIV-1 was supported by two ex vivo experiments from HIV infected aviremic patients. Similarly, the effect of romidepsin on HIV reactivation as found in a drug screen using the T_CM_ model was confirmed in an ex vivo model using patient derived memory and resting CD4 + T-cells [13].

Increases in the p24 content due to reactivation from post-integration latency occur rapidly. However, increases in p24 due to viral replication are comparatively slower, because each replication cycle requires entry, reverse transcription and integration. Consequently, biases in experimental measurements will be smaller at shorter readout times (e.g. 24 hours post reactivation), compared to longer times (e.g. 72 hours post reactivation).

## Conclusions

Replication competent viruses generated in simple co-transfection systems may impact biosafety and bias research results. Researchers should provide evidence proving the replication incompetence of new constructs. The findings impact earlier notions arising from the T_CM_ model and therefore the virus initially included as part of this model cannot be used as originally described [1]. However, the model may still be suitable to understand HIV-1 latency provided that specific modifications are introduced. There are two possible options here. Efforts can be made to avoid recombination by implementation of envelope genes originating from non-HIV species (e.g. VSV-G) [14], by utilizing split vector systems such as these used in gene therapy or more elaborate co-transfection systems with a proven lack of recombination. Alternatively, the model can be used with replication competent viruses in combination with antiretroviral drugs in order to limit the spreading infection. Finally, we would like to state for the record that we are confident that cultured T_CM_ generated as described by Bosque and Planelles [1] continue to be a relevant and suitable cell model for investigations of HIV infection and latency/reactivation, and that the findings described herein do not impact the methods for the generation of such cells or their use in the laboratory.

## Additional file

Additional file 1:
**Experimental results supporting the recombination and excluding laboratory contamination as a source of replication competent virus in the Tcm model and materials and methods related to the study.**
