# Supplementary material for: Replication competent virus as an important source of bias in HIV latency models utilizing single round viral constructs
Source: Retrovirology. 2014 Aug 21;11:70. doi: 10.1186/s12977-014-0070-3 (PMC4156640; doi:10.1186/s12977-014-0070-3)
Supplement: Additional file 1: — Experimental results supporting the recombination and excluding laboratory contamination as a source of replication competent virus in the Tcm model and materials and methods related to the study. [file 12977_2014_70_MOESM1_ESM.docx]

**Replication competent virus as an important source of bias in HIV latency models utilizing single round viral constructs**

Bonczkowski Pawel^a^*, De Spiegelaere Ward^a^*, Bosque Alberto^b^, White Cory H^c^., Van Nuffel Anouk^d^, Malatinkova Eva^a^, Kiselinova Maja^a^, Trypsteen Wim^a^, Witkowski Wojciech^d^, Vermeire Jolien^d^, Verhasselt Bruno^d^, Martins Laura^b^, Woelk Christopher H^e^., Planelles Vicente^b^, Vandekerckhove Linos^a^

1. HIV Translational Research Unit, Department of Internal Medicine, Ghent University and University Hospital, Ghent, Belgium
2. Division of Microbiology and Immunology, Department of Pathology, University Of Utah School of Medicine, Emma Eccles Jones Medical Research Building, Salt Lake City, UT 84112, USA
3. Department of Medicine, University of California, USA
4. Department of Clinical Chemistry, Microbiology and Immunology, Ghent University, Ghent, Belgium
5. Faculty of Medicine, University of Southampton, United Kingdom

* - equal contribution

**Additional file 1: Supplementary data**

**Results**

**High variability between repeated experiments with the TCM model**

Two envelope defective HIV-1 viruses were produced by co-transfection of plasmids encoding a HIV-1 LAI [1] envelope (pLET-LAI) and an envelope-deficient HIV-1 genome – DHIV [1] or NL4.3-IRES-HSA-E*- (Figure S1A, S1C and S1D) and subsequently used to infect cultured TCM cells. Seven days after infection, cells were stimulated with anti-CD3/CD28 microbeads or PHA to reactivate HIV from latency and readout of viral p24 was performed 3 days after activation by flow cytometry [1]. The integrase inhibitor raltegravir was added to the cultures 24 h prior to reactivation, in order to ensure that the increase in p24 production originated from post-integration latency, and not from viral DNA newly integrated upon stimulation.

The outcomes of different experiments involving blood donors as well as different viral productions showed inherent, expected variation, due to variability in the titer of different virus stocks and also donor to donor variation. Some experiments represented very low levels of active infection ranging from 1% to 5% and high degree of latency reaching up to 60-70% (Figure S2A – S2C), revealed upon cell activation with anti-CD3/CD28 beads or PHA. However, approximately 80% of experiments showed high levels of background active infection (20-50%), high levels of cell death and little to no increase in positive signal upon activation as measured by flow cytometry (Figure S2D – S2F).

**Spreading infection in *env*-deficient HIV-1 infected Jurkat E6.1 and SupT1 cells**

To further investigate the high levels of active infection that were frequently observed in the TCM model, Jurkat E6.1 cells were infected with the NL4.3-IRES-HSA-E* vector complemented with pLET-LAI. Intracellular p24 staining was performed to assess infection levels by flow cytometry. The infection levels increased over time, starting from 2.4% at day 3 post-infection reaching up to 60% at day 14 (Figure S3A). The increasing fraction of p24-positive cells over time suggests that a replication competent virus was present in the culture. If that were the case, supernatants from such a culture (primary infection), when applied to fresh cells, should again result in infection (secondary infection). To verify the existence of replication competent virus, culture supernatant from the primary infection was collected, centrifuged to remove cells and debris and applied on fresh Jurkat E6.1 using spinoculation. This secondary infection in Jurkat E6.1 cells reached the level of 27.8% of infection at day 3 (data not shown).

To investigate whether recombination takes place with other *env* deficient viral constructs generated with pLET-LAI, additional batches of vectors were produced in 293T cells: NL4.3-IRES-HSA-E* + pLET-LAI, DHIV alone and DHIV + pLET-LAI. These vectors were used to transduce Jurkat E6.1 cells. The readout performed 3 days after transduction with pLET-LAI complemented NL4.3-IRES-HSA-E* and DHIV revealed levels of 2.9% and 0.8%, reaching 30.8% and 24.9% at day 11, respectively. No transduction was established with DHIV alone (Figure S3B). To exclude contamination of pLET-LAI stock with plasmids containing replication competent full length HIV DNA, a transfection with this construct alone was performed. No virus was produced and transduction with the supernatant did not result in p24 positive cells at any of the measured time points (Figure S3B).

Supernatant from these cultures was added to a new culture of Jurkat E6.1 cells and the levels of infection were measured 4 days later. Rates of 19.8% with the NL4.3-IRES-HSA-E* + pLET-LAI and 20.4% for DHIV+ pLET-LAI indicated the occurrence of a recombination event. No transduction occurred with DHIV alone or pLET-LAI alone (Figure S3C). These findings were confirmed with a number of viral supernatant productions as well as using another cell line – SupT1. A final confirmation was performed on total primary CD4+ T cells (Figure S3D). Interestingly, infectivity of different batches varied between viral productions. This indicates that the recombination is stochastic and its influence on the outcome of the experiment cannot be accurately predicted (Figure S4).

**PCR confirmation of the presence of recombinant virus**

NL4.3-IRES-HSA-E* contains a full length *env* gene except for a 2-base frameshift mutation, while the sequence of *env* in DHIV has a 580 bp deletion within the gp120 coding region to prevent the envelope from being assembled after infection. Primer pairs to confirm the deletion in the DHIV constructs were developed. The ENV primer pair anneals in the common fragment of NL4.3-IRES-HSA-E* and DHIV, DEL primer pair amplifies the sequence of *env* that is missing in the DHIV construct (Figure 1A).

The electrophoretic analysis of the PCRs performed on NL4.3-IRES-HSA-E* and DHIV plasmids with these primer pairs revealed that the ENV primer pair amplified the target sequence, while DEL primer pair worked only in NL4.3-IRES-HSA-E* prep as detected by endpoint PCR (Figure 1B). This demonstrates that the deletion in DHIV is indeed present.

Subsequently, PCR was performed on DNA isolated from infected cells with the ENV and DEL primer pairs to verify whether the *env* deletion had been maintained. The DNA isolation was performed 7 days after transduction. Consistent with plasmid PCR, ENV primer pair amplified a fragment from cells infected with NL4.3-IRES-HSA-E* with pLET-LAI and DHIV with pLET-LAI. As expected, no amplification product was generated with this primer pair on DNA from cells spinoculation with vector DHIV alone, which did not result in p24 expression. This also indicates that the positive signal detected in the other PCR reactions was derived from integrated viral DNA and not from contaminating plasmids that may be present in the viral supernatant after transfection. Primer pair DEL led to amplification of target sequences in the same samples positive in the ENV PCR. The positive signal in cells infected with a virus generated with *env*-deficient DHIV ­+ pLET-LAI indicates the presence of a full length *env* sequence in the viral DNA (Figure 1C), which could only be accounted for by a recombination event.

**Confirmation of the presence of recombinant virus by next generation sequencing**

To further investigate the reconstitution of the full length *env* between DHIV and pLET-LAI, the supernatant from infected cultured central memory T cells derived from 4 donors was collected 10 days after the infection and total RNA-Seq analysis was performed. Paired-end reads were mapped to the HIV genome and showed that RNA was being expressed throughout the entire viral genome including the deleted *env* region (Figure 1D). Reads mapping to the deleted *env* region failed to map anywhere else in the HIV or human (hg19) genomes (data not shown). The presence of reads spanning the region of the deletion in DHIV *env* indicates that an intact sequence of *env* was restored in the construct (Figure 1D – 1F). In summary, RNA-Seq analysis provided strong evidence of active HIV replication in the latent TCM model and clearly showed that full length *env* mRNA is being present.

**Infection with VSV-G pseudotyped viruses failed to produce recombinant viruses**

HIV viral particles produced from *env*-deficient backbones DHIV or NL4.3-HSA pseudotyped with a non-HIV derived envelope protein, i.e. VSV-G protein were produced to test if replication competent virus would be generated. The levels of infection of Jurkat E6.1 cells with these 2 viruses remained at a constant level throughout the experiment indicating that recombination happens between homologous HIV sequences only (Figure S5A). This finding was confirmed by performing a secondary infection with supernatant collected from infected cells. No infection was established in these circumstances (Figure S5B-S5E). The different origin and sequence of VSV-G *env* probably prevents recombination with the HIV-based backbone plasmid. These data provides further evidence that replication competent virus only is generated by the co-transfection of an HIV derived full length *env* containing plasmid with an *env* deleted HIV containing plasmid.

**A large deletion in *env* reduces, but does not eliminate the generation of replication competent recombinant virus**

To investigate if the size of the deletion in the *env* gene influences the frequency of recombination, a new proviral construct derivative was engineered containing a larger deletion in *env*. In contrast to DHIV, where the deletion is approximately 600 bp long, in this new construct, referred to as DDHIV, an additional 800 bp were deleted (Figure S1B). A batch of pLET-LAI complemented vector was generated and used to infect SupT1 cells in parallel with the same titer of pseudotyped DHIV. 6 hours after infection, cells were trypsinized to eliminate input virus. 4 days later, supernatant from these infected cultures was used for secondary infection of MT-2 cells, in order to test for the presence of replication competent recombinants. Flow cytometry readout performed at day 2 after infection revealed that a secondary infection was established with both constructs, however, the rate of infection was lower for DDHIV + pLET-LAI than for DHIV + pLET-LAI (Figure S6). This indicates that the size of deletion is inversely correlated with the generation of replication competent virus, but it equally indicates that a bigger deletion does not prevent the generation of these replication competent virions.

**Methods**

**Plasmids**

NL4-3-IRES-HSA-E* was constructed by excising the deficient *env* sequence from the pBR NL4-3 Nef+ IRES eGFP [2] vector (kindly provided by Dr. F. Kirchhoff, Institute of Virology, University of Ulm, Ulm, Germany) using AgeI and NpaI restriction enzymes (New England Biolabs). This deficient *env* sequence contains a two base frameshift mutation at the level of the NdeI restriction site. Subsequently, the functional *env* sequence from pNL4.3-HSA-IRES [3] (kindly provided by Dr. M.J. Tremblay, Faculté de Médecine, Université Laval, Québec, Canada) was replaced by a restriction at the AgeI and NpaI and a ligation with the deficient *env* sequence from the pBR NL4-3 Nef+ IRES eGFP plasmid. The product was transformed in DH5 alpha bacteria.

Generation of the DHIV plasmid has been previously described [4]. In short, a fragment between two BglII restriction endonuclease sites located at nucleotides 7032 and 7612 in the HIV-1 NL4-3 sequence was cut and the ends re-ligated. This generated a 580 base-pair deletion within the gp120-coding region, and rendered the downstream portion of the gene out of frame.

pLET-LAI construct was generated as previously described [5]. In short, complete LTR, *tat* and *env* genes from HIV-1 were inserted into plasmid pUC18. The SalI-XhoI fragment containing *env* was ligated downstream of BglII-NarI fragment containing the LTR sequence. The noncoding AvaI-BglII fragment from hepatitis B virus was ligated 3’ to the *env* gene to provide for poly(A), splicing acceptor sequences and termination sequences.

The DDHIV construct with approximately 1400 bp deletion in *env* was constructed by introducing 2 NotI restriction sites by QuickChange site-directed mutagenesis. The site positioned after Vpu ORF was generated with the following primers: forward 5’- CATAATAGACTGTGACCCACAATTTTGC**GG**C**C**G**C**ACTACAGATCATCAATATCCCAAG-3’ and reverse 5’- CTTGGGATATTGATGATCTGTAGT**G**C**G**G**CC**GCAAAATTGTGGGTCACAGTCTATTATG-3’. This resulted in changing the original sequence from GCTACAGAA to GCGGCCGCA. The site before RRE was generated with the following primers: forward 5’-CCCACTGCTCTTTTTTCTCTCG**C**GG**CCGC**TCTTCTCTTTGCCTTGGTGG-3’ and reverse 5’-CCACCAAGGCAAAGAGAAGA**GCGG**CC**G**CGAGAGAAAAAAGAGCAGTGGG-3’. This resulted in changing the original sequence from GTGGTGCA to GCGGCCGC. The fragment between these restriction sites was subsequently cut and the ends re-ligated. The stop codon was introduced after RRE with the following primers: forward 5’-AGGATCAACAGCTCCTG**TGA**ATTTGGGGTTGCTCTGG-3’ and reverse 5’-CCAGAGCAACCCCAAAT**TCA**CAGGAGCTGTTGATCCT-3’. This led to a change in the original sequence from CTGGGGATT to CTGTGAATT.

To exclude the possibility of contamination of glycerol stocks used to grow the plasmids, all plasmids were single-colony purified. A restriction digest was performed and successful cloning was confirmed by performing electrophoretic trace analysis on the restriction digest of all plasmids used in the study.

**Transfection**

Virus stocks were prepared by calcium phosphate transfection of 293T cells (DZSM, Braunschweig, Germany) according to manufacturer’s instructions (Life Technologies). 7.5 x 10^5^ 293T cells were seeded in 6 cm plates in 6 ml of IMDM medium (Life Technologies, Merelbeke, Belgium) supplemented with 10% FCS (Hyclone, Thermofisher Scientific, Waltham, MA, USA), L-glutamine and antibiotics (Life Technologies) 24h prior to transfection. 1 h before transfection, solutions consisting of 8 µg backbone plasmid and 2 µg envelope plasmid for complemented vectors or 10 µg backbone plasmid only were prepared. The medium was replaced 24h after transfection with fresh IMDM medium and the virus-containing supernatants were collected after another 24h incubation. The supernatant was briefly centrifuged at 600 g to remove cells and debris, aliquoted and frozen at -80°C.

**Cell isolation and culture**

# Peripheral blood mononuclear cells (PBMCs) were isolated following density gradient centrifugation. Blood from healthy donors was diluted in PBS (Lonza, Verviers, Belgium) at a ratio of 1:1, 25 ml of diluted blood was slowly added on top of 12 ml **Lymphoprep^TM^** (Axis-Shield, Oslo, Norway), centrifuged at 770 g for 20 min at room temperature. Isolated PBMCs were washed twice in PBS 2 and naïve CD4+ T cells or whole CD4+ T cells were isolated using the Naive CD4^+^ T Cell Isolation Kit II, human or CD4^+^ T Cell Isolation Kit II, respectively (MiltenyiBiotec, Bergisch Gladbach, Germany). This microbead-based negative selection sorting results in highly pure populations exceeding 95% purity as analysed by flow cytometry (data not shown).

# Primary CD4+ T-cells were routinely cultured in a 5% CO_2_ incubator in 96-well plates at a concentration of 1 million/ml of RPMI medium (Invitrogen) supplemented with 10% FCS, L-glutamine, antibiotics and IL-2 (30IU/ml) (Peprotech, Rocky Hill, NJ, USA). Jurkat E6.1 (ATCC Cell Biology Collection, Manassas, VA, USA), SupT1 (Cat. No 100 obtained through the NIH AIDS Reagent Program, Division of AIDS, NIAID, NIH from Dr. Dharam Ablashi) and MT-2 (Cat. No 237 obtained through the NIH AIDS Reagent Program, Division of AIDS, NIAID, NIH from Dr. Douglas Richman) cell lines were cultured in a 7% CO_2_ incubator in IMDM medium (Invitrogen) supplemented with 10% FCS, L-glutamine and antibiotics. Medium change was performed every 2 or 3 days for primary cells and every 3 or 4 days for cell lines and was also aimed at removing most of the residual virus from the culture after infection. The use of cell lines enabled long term tracking of infection levels to investigate the phenomenon of increasing active infection.

# *In vitro* differentiated central memory T cells (TCM) were generated as previously described [6]. Briefly, naïve CD4+ T cells were cultured for 3 days in non-polarising conditions, i.e. RPMI supplemented with 1 µg/ml anti-IL-4, 2 µg/ml anti-IL-12, 10 ng/ml TGF-β (all Peprotech) and anti-CD3/CD28 microbeads (Invitrogen). After these three days, magnetic beads were removed with DynaMag™ Spin Magnet (Life Technologies), cells were collected, counted and resuspended in fresh RPMI with 30IU/ml IL-2 at 1 million cells in 1 ml of RPMI medium before seeding in 96 well plates. Daily medium change was performed for 4 additional days. The central memory T cells were infected at day 7 post-isolation.

**Primary and secondary infections**

Cells were infected by spinoculation in flat bottom 96-well plates. Target cells were distributed at 2 x 10^5^ cells/well, viral supernatant was added at a 20 ng p24 per well for cell lines or 100 ng p24 per well for primary cells as quantified by the RT assay and converted to p24 values as previously described [7], mixed and spinoculated for 90 min, 710 g, 32°C. After spinoculation, the supernatant containing HIV was replaced with fresh RPMI or IMDM medium for primary cell cultures or cell lines respectively. Cells were transferred to U-bottom 96-well plates for further culture.

Reinfections were performed by brief centrifugation (600 g) of supernatants from previously infected cells to remove cell debris and mixing them with non-infected cells prior to spinoculation in the same conditions.

**Cell activation**

# Cell activation was performed to reveal post-integration latency in the TCM model. 1 day prior to activation, cells were distributed at 2 x 10^5^ per well. To differentiate between pre- and post-integration latency, a fraction was treated with raltegravir at 500 nM (Cat. No 11680 from Merck & Company, Inc.) obtained through the NIH AIDS Reagent Program, Division of AIDS, NIAID, NIH). Cells were stimulated with PHA (1 µg/ml) or anti-CD3/CD28 microbeads (Dynabeads® Human T-Activator CD3/CD28, Invitrogen) for 72 h before the readout by flow cytometry.

**PCR analysis of plasmid and cellular DNA**

Sequences of NL4.3-IRES-HSA-E* and DHIV were aligned in Vector NTI® software (Life Technologies). NL4.3-IRES-HSA *env* gene contains the full sequence; DHIV *env* gene contains a large 580 bp deletion. Two PCR primer pairs were designed: ENV that amplifies the common fragment of *env* (ENV forward 5’-TACCTGTGTGGAAGGAAGCAACCA-3’, ENV reverse 5’-CACATGGCTTTAGGCTTTGATCCC-3’) and DEL that amplifies the deleted fragment of *env* in DHIV (DEL forward 5’-GGGACCCAGAAATTGTAACGCACA-3’, DEL reverse 5’-GCATGGGAGTGTGATTGTGTCACT-3’).

These primers were used to detect target sequences in plasmid DNA isolated from bacterial glycerol stock using QIAprep Spin Miniprep Kit (Qiagen, Venlo, Netherlands) or cellular DNA isolated from infected Jurkat E6.1, SupT1 or PBL cells with DNeasy Blood & Tissue Kit (Qiagen) following manufacturer’s protocol. Purified DNA was next subjected to endpoint PCR using GeneAmp® PCR System 9700 (Applied Biosystems, Life Technologies). 20 µl reaction mix consisted of 1x buffer, 0,12 mM dNTPs, 2 mM MgCl_2_, 2 U Platinum Taq polymerase (all Invitrogen), water (Sigma-Aldrich, Diegem, Belgium) and 250 nM forward and reverse primers.

PCR reaction settings were: 95°C for 5 min, 40 cycles of 95°C for 30 s, 60°C for 30 s, 72°C for 30 s and finally 72°C for 5 min. After the reaction, the samples were analysed by electrophoretic separation using LabChip® GX (PerkinElmer, Waltham, MA, USA).

**P24 staining and flow cytometry**

Intracellular p24 staining was performed to quantify levels of HIV infected cells. 1 x 10^5^ cells were transferred to a V-bottom 96 well plate and spinoculated for 5 min, 380 g, at room temperature to remove the medium. Next, 150 µl of PBS was added before another spinoculation. The buffer was aspirated, cells suspended in 30 µl Reagent A (Fix&Perm, ADG, Vienna, Austria) and incubation of 15 min followed. Afterwards, 150 µl of PBS was added and another spinoculation was performed. The buffer was collected and the cells resuspended in 30 µl Reagent B (Fix&Perm, ADG) with 1 µl anti-p24 monoclonal antibody AG3.0 (Cat. No 4121) obtained through the NIH AIDS Reagent Program, Division of AIDS, NIAID, NIH originally from Dr. Jonathan Allan). After 30 min of staining, the primary antibody was washed away and the cells were suspended in 100 µl PBS with 1:200 Alexa Fluor 488 goat anti-mouse IgG (H+L) antibody (Invitrogen). After additional 15 min of staining, the cells were spinoculated and suspended in PBS after removing the antibody solution to be analysed by flow cytometry using FACSCalibur (BD) and Flowing Software 2.5.0 (Perttu Terho, Turku, Finland).

**NGS data generation and analysis**

Total RNA was isolated from the TCM models derived from 4 healthy donors and deemed of sufficient quality for Total RNA-Seq analysis (RNA integrity number >7 as assessed by Agilent Bioanalyzer 2100, Agilent*,* Santa Clara, CA, USA). For Total RNA-Seq analysis, Ribozero was used to deplete ribosomal RNAs and the remaining RNA was DNase treated and prepared using the Illumina TrueSeq^TM^ RNA Sample Preparation Kit for 50 bp paired-end sequencing with the Illumina HiSeq 2000. Resulting reads were mapped to the HIV genome using TopHat [8] and counted using HT-Seq (http://www-huber.embl.de/users/anders/HTSeq/) for visualization using the Integrative Genome Viewer (IGV) [9] to produce a coverage map of reads along the entire HIV genome.

**Acknowledgements**

The following reagents were obtained through the AIDS Research and Reference Reagent Program, Division of AIDS, NIAID, NIH: Raltegravir (Cat # 11680) from Merck & Company, Inc., Monoclonal Antibody to HIV-1 p24 AG3.0 (Cat # 4121) from Dr. Jonathan Allan, MT-2 cell line (Cat # 237) from Dr. Douglas Richman and SupT1 cell line (Cat # 100) from Dr. Dharam Ablashi.

The authors acknowledge the generous gift of the pBR NL4-3 Nef+ IRES eGFP vector provided by Dr. F. Kirchhoff, Institute of Virology, University of Ulm, Ulm, Germany and the pNL4.3-HSA-IRES vector provided by Dr. M.J. Tremblay, Faculté de Médecine, Université Laval, Québec, Canada.

**Illustrations and figures**

**
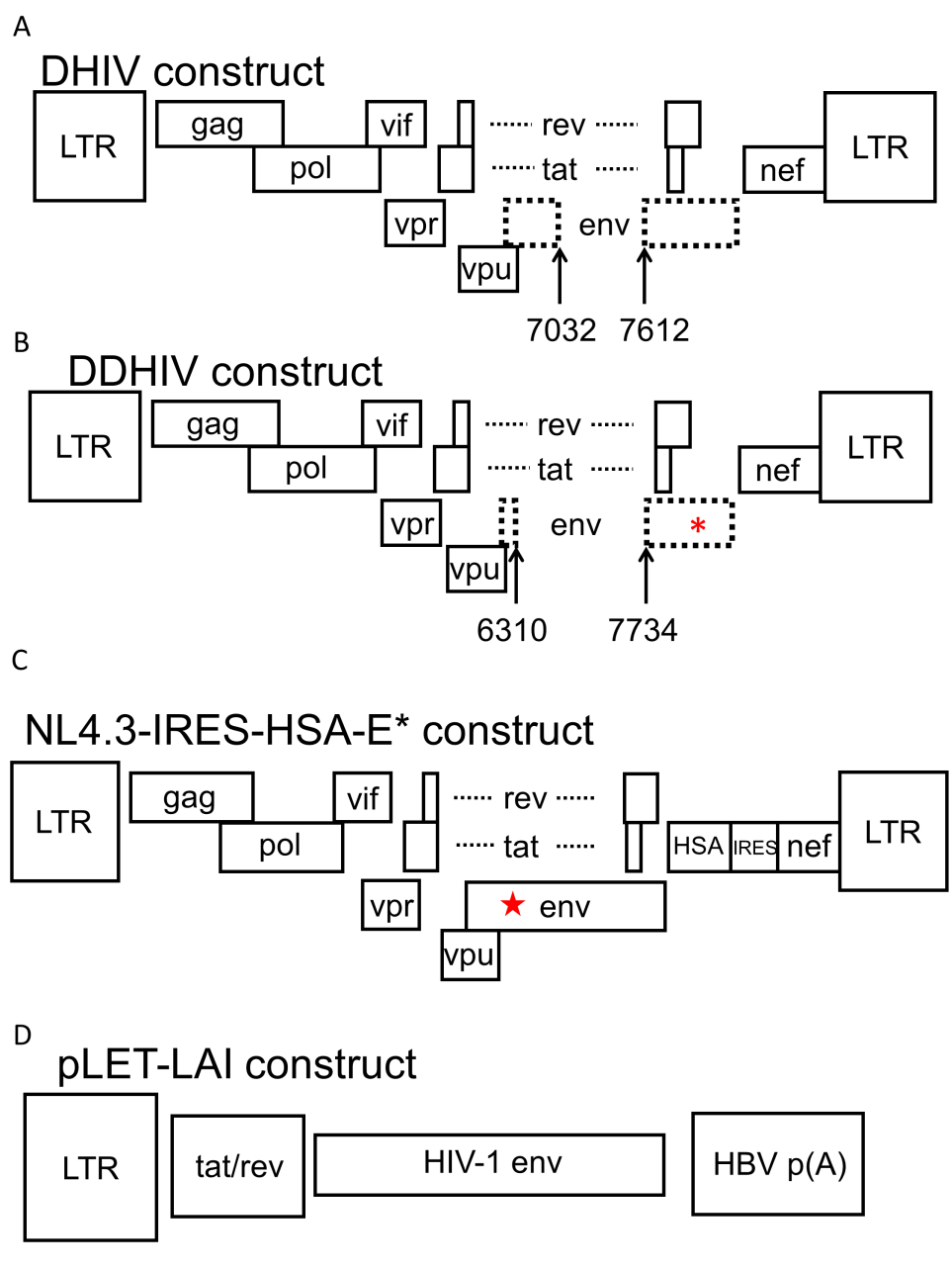
**

**Figure S1. Graphical representation of constructs used in this study.** Dotted lines in DHIV **A.** and DDHIV **B.** represent the truncated *env* gene. The arrows and nucleotide numbers indicate the positions of these deletions with reference to HIV-1 NL4.3 sequence. The asterisk represents a STOP codon introduced after RRE sequence. **C.** NL4.3-IRES-HSA-E* construct. The star represents the frameshift mutation in the envelope gene. **D.** pLET-LAI construct containing a full length *env* sequence.


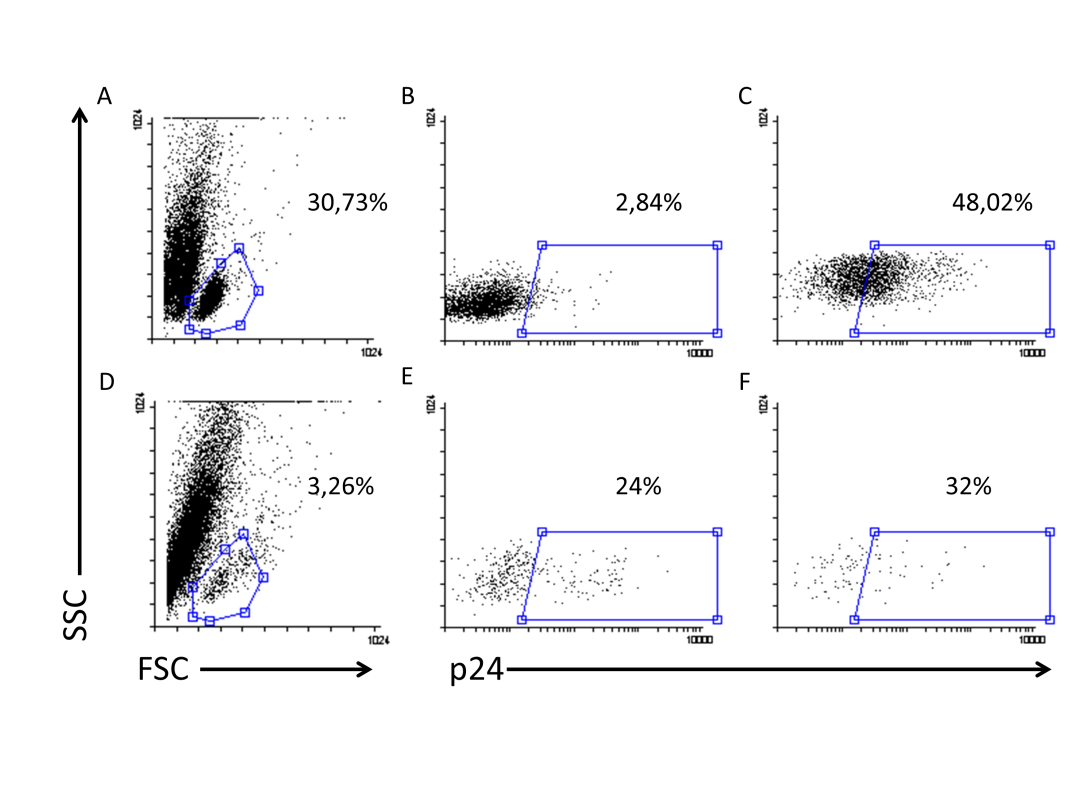


**Figure S2. Active infection levels in the TCM model are highly variable and can be independent of cell activation.** Dot plots show cell survival upon transduction with DHIV + pLET-LAI as determined by side scatter (SSC) and forward scatter (FCS) and p24 expression levels in function of side scatter. The percentages indicate p24 positive cells in the selected region. **A-C.** An experiment with normal cell survival **(A),** low background active infection **(B)** and high levels of p24 expression upon activation representing reactivated latently infected cells **(C)**. **D-F.** An experiment with high cell death **(D)**, high active infection in the absence of cell activation **(E)** and little difference in p24 expression as determined by flow cytometry upon activation **(F)**.


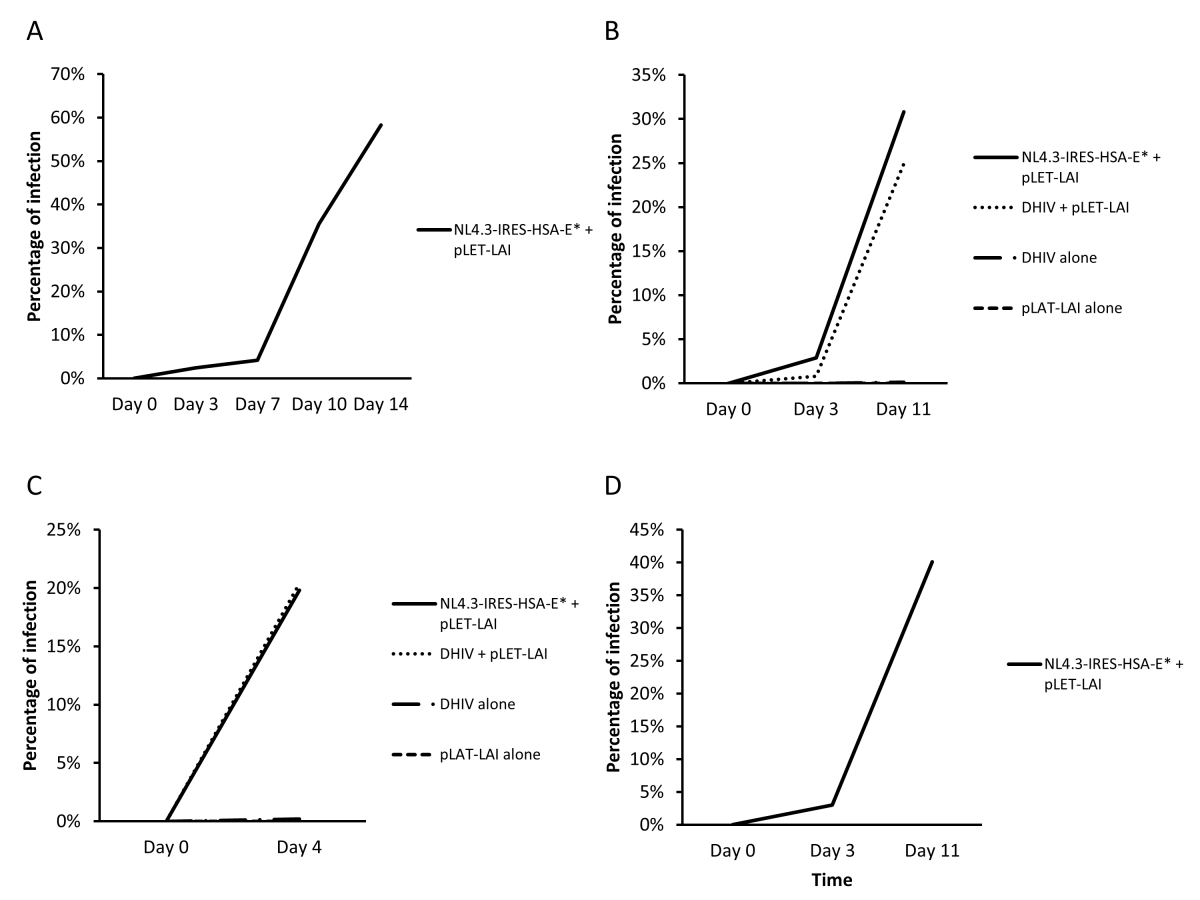


**Figure S3. Increasing percentages of HIV infected cells over time in different experiments using *env* deficient viral constructs co-transfected with pLET-LAI as measured by intracellular p24 staining and flow cytometry readout**. **A**. Primary infection of Jurkat E6.1 cells with NL4.3-IRES-HSA-E* + pLET-LAI. **B**. Primary infection of Jurkat E6.1 with different constructs: NL4.3-IRES-HSA-E* + pLET-LAI, DHIV + pLET-LAI, DHIV alone, pLET-LAI alone. **C**. Secondary infection of Jurkat E6.1 cells performed with supernatants from the infected cells described in B. **D**. Primary infection of total CD4+ T cells with NL4.3-IRES-HSA-E* + pLET-LAI.


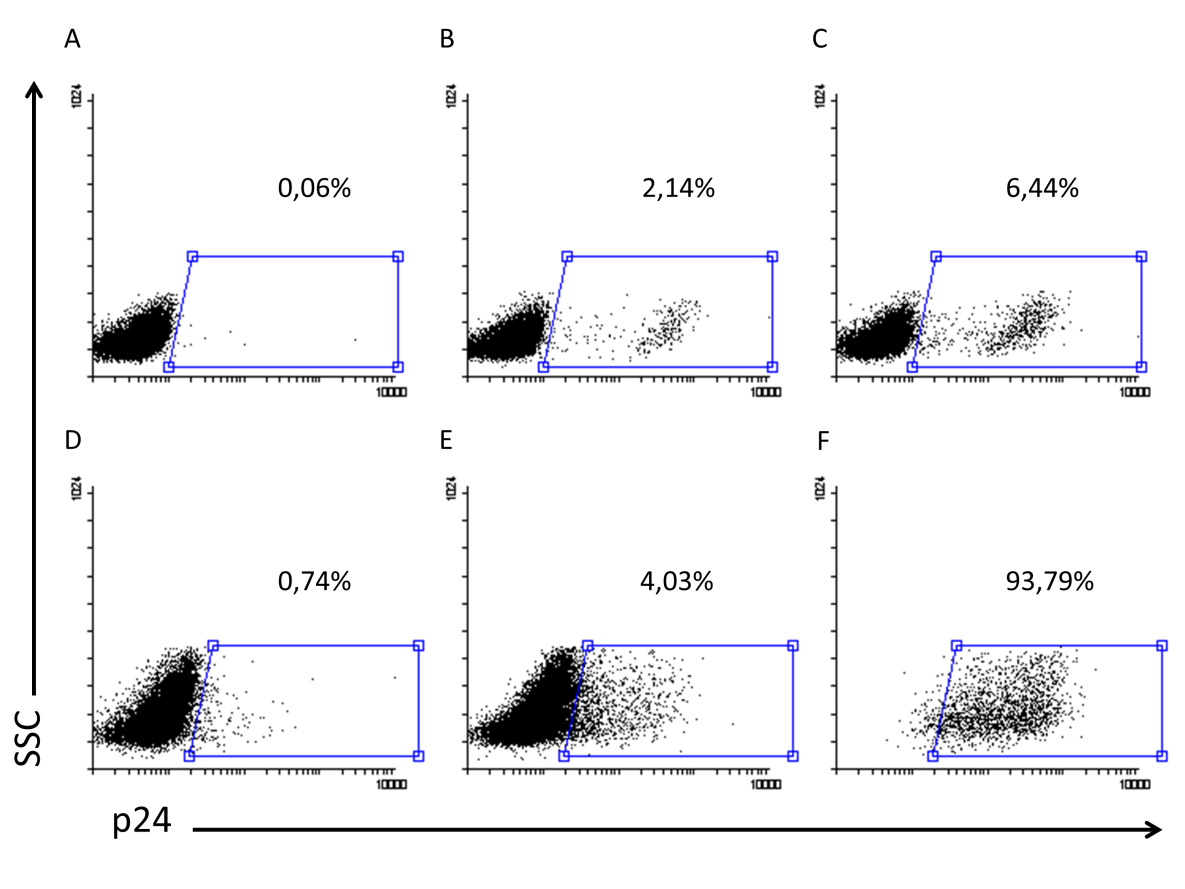


**Figure S4. Percentage of cells infected with DHIV + pLET-LAI originating from different viral productions as measured by p24 staining and flow cytometry readout shows differences between particular virus productions**. **A-C.** Primary infection of SupT1 cells leads to infection establishment, with different kinetics depending on viral production. **A.** Mock infection. **B.** Primary infection with DHIV + pLET-LAI, batch 1. **C.** Primary infection with DHIV + pLET-LAI, batch 2. **D-F.** Secondary infection of MT2 cells with supernatants from cultures described in A-C reveals differences between viral productions in terms of infectivity. **D.** Mock infection. **E.** Secondary infection with DHIV + pLET-LAI, batch 1. **F.** Secondary infection with DHIV + pLET-LAI, batch 2.


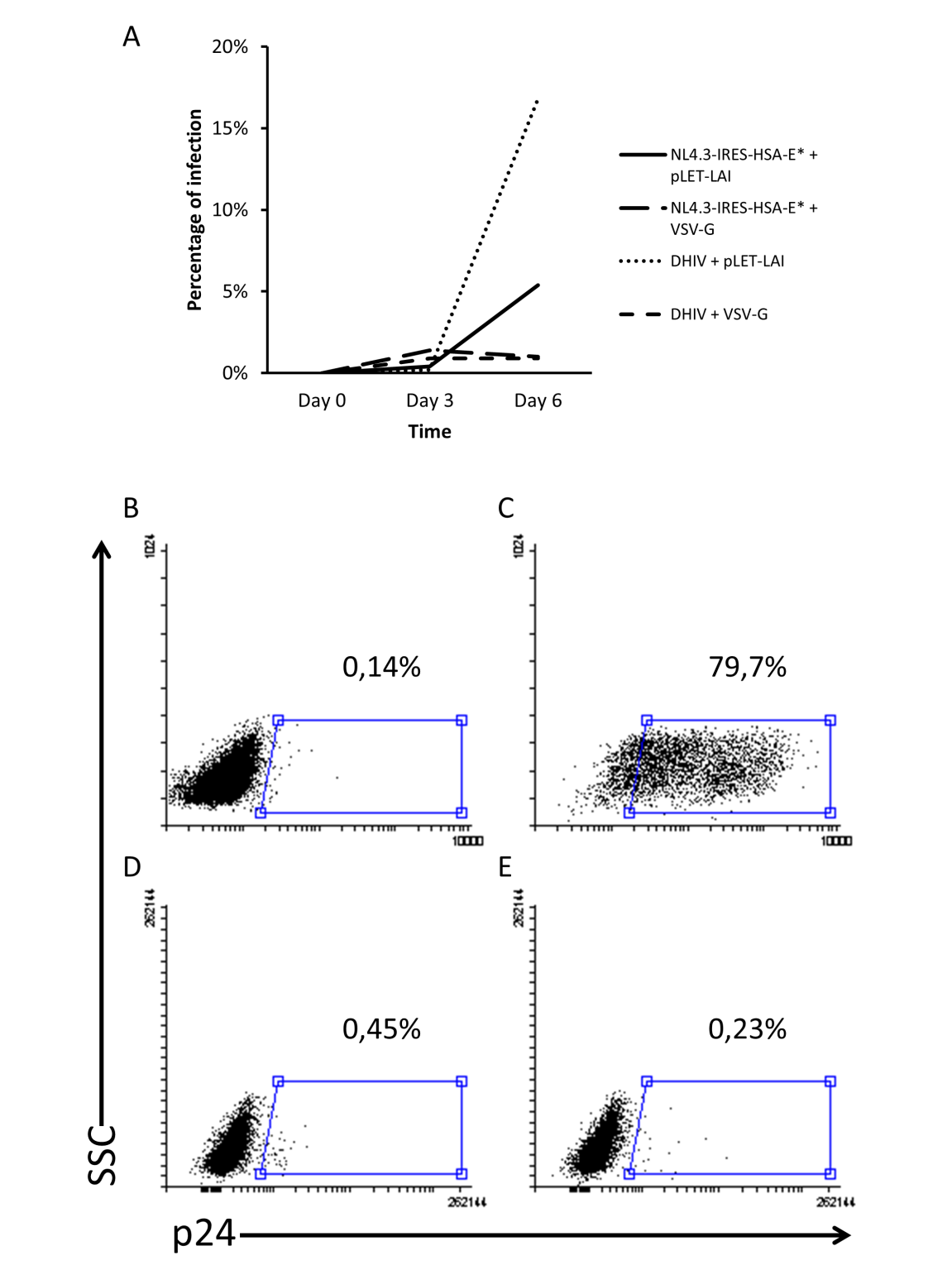


**Figure S5. Percentage of cells infected with pLET-LAI or VSV-G complemented constructs over time.** **A.** Infection levels with vector generated by co-transfected with pLET-LAI (NL4.3-IRES-HSA-E* + pLET-LAI and DHIV + pLET-LAI) increases over time, while VSV-G pseudotyping (NL4.3-IRES-HSA-E* + VSV-G and DHIV + VSV-G) leads to stable levels of transduction. **B-D.** Percentage of cells infected with DHIV pseudotyped with VSV-G as measured by p24 staining and flow cytometry readout shows that recombination between HIV backbone and VSV-G envelope does not occur. **A-B.** Primary infection of SupT1 cells with DHIV pseudotyped with VSV-G leads to establishment of infection. **A.** Mock infection. **B.** Infection with DHIV + VSV-G. **C-D.** Secondary infection with the supernatant from infected SupT1 cells shown in A-B performed on MT2 cells. **C.** Mock infection. **D.** Infection with supernatant from SupT1 cells infected with DHIV + VSV-G.


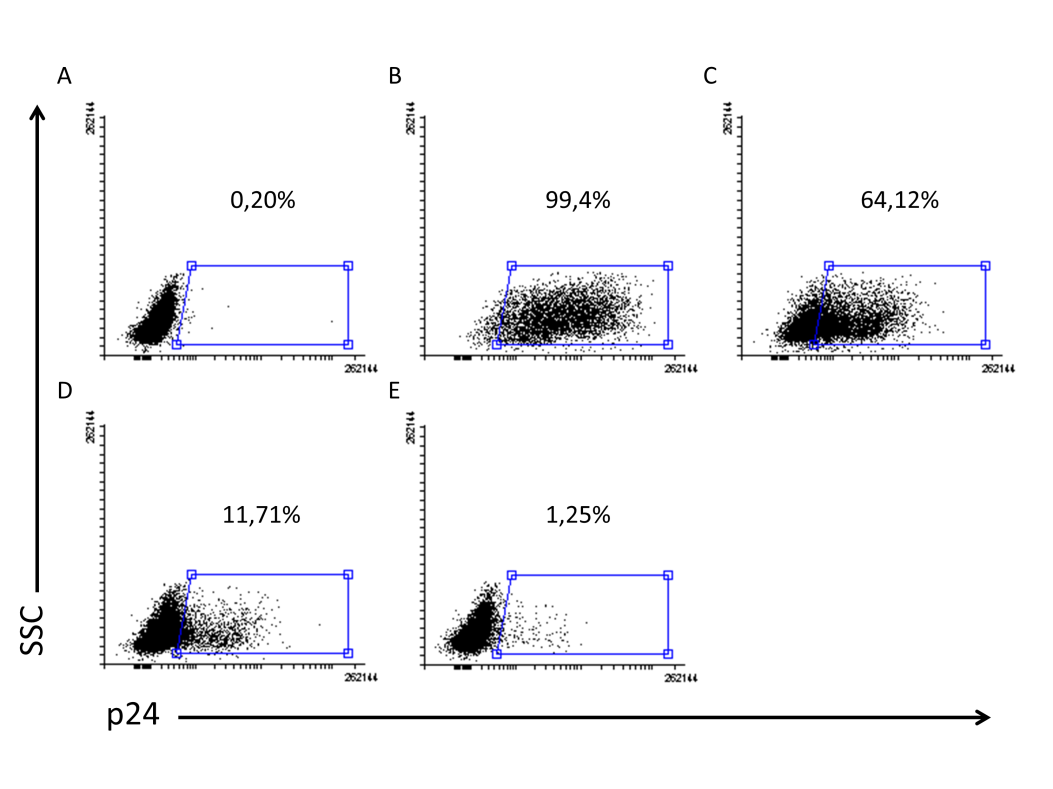


**Figure S6. Percentage of MT-2 cells transduced with DHIV + pLET-LAI or DDHIV + pLET-LAI during secondary infection shows varying extent of recombination between the HIV backbone and *env* gene supplied upon co-transfection.** **A**. Mock infection. **B**. Infection with DHIV + pLET-LAI. **C**. Infection with DHIV + pLET-LAI, 10% of the titer used in B. **D**. Infection with DDHIV + pLET-LAI. **E**. Infection with DDHIV + pLET-LAI, 10% of the titer used in D.

**References**

1. Bosque A, Planelles V: **Induction of HIV-1 latency and reactivation in primary memory CD4(+) T cells.** *Blood* 2009, **113:**58-65.

2. Wildum S, Schindler M, Munch J, Kirchhoff F: **Contribution of Vpu, Env, and Nef to CD4 down-modulation and resistance of human immunodeficiency virus type 1-infected T cells to superinfection.** *J Virol* 2006, **80:**8047-8059.

3. Imbeault M, Lodge R, Ouellet M, Tremblay MJ: **Efficient magnetic bead-based separation of HIV-1-infected cells using an improved reporter virus system reveals that p53 up-regulation occurs exclusively in the virus-expressing cell population.** *Virology* 2009, **393:**160-167.

4. Andersen JL, DeHart JL, Zimmerman ES, Ardon O, Kim B, Jacquot G, Benichou S, Planelles V: **HIV-1 Vpr-induced apoptosis is cell cycle dependent and requires Bax but not ANT.** *PLoS Pathog* 2006, **2:**1106-1119.

5. Langlade-Demoyen P, Michel F, Hoffenbach A, Vilmer E, Dadaglio G, Garicia-Pons F, Mayaud C, Autran B, Wain-Hobson S, Plata F: **Immune recognition of AIDS virus antigens by human and murine cytotoxic T lymphocytes.** *J Immunol* 1988, **141:**1949-1957.

6. Bosque A, Planelles V: **Studies of HIV-1 latency in an ex vivo model that uses primary central memory T cells.** *Methods* 2011, **53:**54-61.

7. Vermeire J, Naessens E, Vanderstraeten H, Landi A, Iannucci V, Van Nuffel A, Taghon T, Pizzato M, Verhasselt B: **Quantification of Reverse Transcriptase Activity by Real-Time PCR as a Fast and Accurate Method for Titration of HIV, Lenti- and Retroviral Vectors.** *PLoS One* 2012, **7:**e50859.

8. Trapnell C, Pachter L, Salzberg SL: **TopHat: discovering splice junctions with RNA-Seq.** *Bioinformatics* 2009, **25:**1105-1111.

9. Robinson JT, Thorvaldsdottir H, Winckler W, Guttman M, Lander ES, Getz G, Mesirov JP: **Integrative genomics viewer***.* *Nat Biotechnol* 2011 Jan;**29**(1):24-6. doi: 10.1038/nbt.1754.
